# Potential Antimicrobial Use of Cannabidiol in Dentistry: A Scoping Review

**DOI:** 10.3390/dj13110519

**Published:** 2025-11-06

**Authors:** Matias Mederos, Alejandro Elizalde-Hernández, Alejandro Francia, Luiz Alexandre Chisini, Cristina Pereira Isolan, Rafael R. Moraes, Rafael Guerra Lund, Carla David

**Affiliations:** 1 Graduate Program in Dentistry, University of the Republic, Montevideo 11600, Uruguay; matiasmederos@odon.edu.uy; 2 Graduate Program in Dentistry, School of Dentistry, Federal University of Pelotas, Pelotas 96015-560, Brazil; aleeh87@outlook.com (A.E.-H.); alexandrechisini@gmail.com (L.A.C.); moraesrr@gmail.com (R.R.M.); rafael.lund@gmail.com (R.G.L.); 3 Department of Dental Biology, University of the Republic, Montevideo 11600, Uruguay; ale-fp@hotmail.com; 4 Department of Dentistry, School of Dentistry, Federal University of Jequitinhonha and Mucuri Valleys, Diamantina 39190-000, Brazil; cristina.isolan@ufvjm.edu.br; 5 Biopathological Research Group, School of Dentistry (GIBFO), University of the Andes Mérida, Mérida 5101, Venezuela

**Keywords:** antibacterial activity, antibiofilm activity, cannabidiol, dental biofilm, dental plaque

## Abstract

**Background/Objectives:** The use of cannabidiol (CBD) as an antimicrobial and antifungal agent has gained interest in medicine, with studies suggesting potential against various microorganisms. However, its effectiveness against oral pathogens remains underexplored in dental research, highlighting the need for further studies. This scoping review summarizes current evidence on the antimicrobial properties of CBD in dental and oral health. **Methods:** A systematic search was conducted across seven databases (PubMed, the Cochrane Library, Scopus, Embase, Web of Science, SciELO, and LILACS) up to January 2025. The inclusion criteria encompassed studies that explored the effects of CBD on oral microbiology (in vitro and in vivo in animal models), regardless of language or year of publication. The gray literature was evaluated in the Google Scholar database. **Results:** A total of 1284 articles were identified, of which 10 met the inclusion criteria for this scoping review. These studies, published between 2019 and 2025, primarily focused on bacterial and fungal cultures. The most commonly used methods were the minimum inhibitory concentration test and counting colony-forming units. The contact methods between CBD and bacterial/fungal cell cultures were either dilution or direct contact. **Conclusions:** CBD shows promising antimicrobial properties against a range of oral bacteria and fungi, suggesting its potential application in managing oral health conditions.

## 1. Introduction

The oral cavity serves as a habitat for a diverse range of pathogens, some of which are also present in other body parts, while others are unique to this region. Over 700 distinct species of microorganisms have been identified in the oral cavity [1]. Oral health is maintained through a delicate balance between host factors and the resident microorganisms [2,3]. However, systemic or local factors, such as diseases, dietary habits, substance use, or poor oral hygiene can affect the immune system [2]. Such imbalances may lead to the development of oral pathologies, including dental caries and periodontal disease, by promoting the overgrowth of oral pathogens (e.g., *Porphyromonas gingivalis*, *Streptococcus mutan*, *Candida albicans*, and others) [4,5].

To avoid or combat the proliferation of oral pathogens, the use of various antimicrobial and antifungal agents has been proposed [6,7]. Among the most widely used antimicrobial agents are cetylpyridinium chloride [8], triclosan [9], and chlorhexidine (the gold standard due to its high efficacy) [10]. As for antifungals, nystatin [11] and fluconazole [12] can be mentioned. However, the use of some of these agents is often associated with undesirable side effects. For instance, antimicrobials may cause dental pigmentation and taste alterations [13,14], while antifungals can disrupt the balance of the host’s microbiota. Consequently, their use is typically restricted to specific clinical scenarios or limited treatment durations [15].

Given these limitations, alternative approaches have been explored in the literature [10,16]. In recent years, plant-based products, such as essential oils, have gained relevance due to their minimal reported side effects [10,16,17]. Cannabinoids have demonstrated efficacy in treating various pathogen-related conditions, including certain fungal strains and skin infections like acne [18]. Specific cannabinoids, such as cannabidiol (CBD), cannabigerol (CBG), tetrahydrocannabinol (THC), and cannabinol (CBN), have been investigated for these purposes [19].

CBD appears to be the cannabinoid most frequently selected as an antimicrobial agent against diverse oral pathogens [20,21]. This preference is likely due to its broad therapeutic profile—including analgesic, anti-inflammatory, neuroprotective, and osteoinductive effects—alongside its low toxicity and lack of psychotropic effects, making CBD an attractive candidate for medical applications. Furthermore, its potential in dentistry has been demonstrated in various studies, suggesting promising prospects for its future use in dental treatments [20,21]. CBD has been evaluated both as a standalone agent [22] and in combination with other known antimicrobials, such as triclosan [23]. It is important to note that the oral strains tested originated from both commercial sources [23] and plaque samples directly collected from individuals’ oral cavities [24].

Despite the promising findings regarding antimicrobial activity, evidence supporting the efficacy of CBD against oral diseases such as caries, periodontitis, and candidiasis, or against diverse oral pathogens, remains limited, especially considering that CBD is one of the most abundant cannabinoids in the plant [20,21]. Therefore, this scoping review aims to analyze and synthesize the available evidence on the use of CBD as an antimicrobial and antifungal agent against oral microorganisms.

## 2. Materials and Methods

### 2.1. Protocol and Registration

This scoping review was reported according to the Preferred Reporting Items for Systematic Reviews and Meta-Analysis Extension for Scoping Reviews guidelines [25]. The study protocol was developed based on the framework proposed by Peters et al. (2015) [26], according to the Joanna Briggs Institute [27], and is available on the Open Science Framework at https://doi.org/10.17605/OSF.IO/GSQWM (accesed on 5 May 2025). The review focused on studies that compared CBD with other substances or treatments, if applicable, particularly in the context of dental and oral medicine. The research question was formulated as follows: “What is the current state of knowledge regarding the microbiological properties of CBD?”.

### 2.2. Criteria Used for the Mapping Process

To identify the important concepts of the primary review question, the PCC framework for scoping reviews (population, concept, and context) was taken into consideration.

Population: In vitro microbial cultures or in vivo animal models investigating the microbiological properties of CBD.

Concept: Microbiological effects, mechanisms, and applications of CBD, including its antimicrobial and antifungal properties.

Context: Dental and oral health, particularly in the prevention or treatment of oral infections and pathologies.

### 2.3. Eligibility Criteria

The inclusion and exclusion criteria used are presented in Table 1.

### 2.4. Information Sources and Search

The search strategy was defined using MeSH terms/descriptors and Boolean operators (AND/OR). This step was collaboratively designed and conducted by all authors of this study prior to registering the protocol in the database. Two trained researchers (M.M. and A.E.-H.) performed independent searches and subsequently cross-checked the number of articles retrieved from each database. Any discrepancies were resolved by a third researcher with expertise in search syntax (C.D.). This process was crucial to ensuring a comprehensive overview of the current state of knowledge regarding the biological and microbiological aspects of CBD. The search strategy was customized for each database due to inherent differences.

The strategy developed for PubMed/Medline, incorporating the terms defined in the first search phase and employing the Boolean operator “OR” (Table 1), was adapted for use in the Cochrane Library, Scopus, Embase, Web of Science, Scielo, and Lilacs databases (Supplementary Table S1). Two trained researchers (C.D. and A.E.-H.) independently performed this search and cross-checked the number of articles retrieved from each database. A third researcher, as detailed in Table 2, resolved any discrepancies. Additionally, a manual search of the reference lists from all selected studies was conducted following the full-text reading phase. The gray literature was evaluated in the Google Scholar database (the first 100 records), with the search and browsing history kept clean to avoid altering the search results. No restrictions regarding language or publication period were applied.

To align the researchers, internal tests were conducted to assess agreement among the reviewers. Additionally, consensus was established through a third party in cases of discrepancies, and an initial pilot test was planned, in which the first 100 cases were reviewed to identify any inconsistencies. Following this stage, a kappa analysis was performed, aiming to achieve a value equal to or greater than 0.61 (Cohen’s K = 0.69) among the researchers, thereby ensuring substantial (0.61–0.80) reliability in the review process.

### 2.5. Selection of Sources of Evidence

Retrieved articles were uploaded to Endnote 21.0.1 software (Clarivate Analytics©, Thomson Reuters, New York, NY, USA), and duplicate records were automatically removed by the software. After the removal of duplicates, a single file containing all the found studies was generated and imported into the Rayyan application (Hamad Bin Khalifa University, Doha, Qatar) [28] for study selection. The same two researchers carried out this selection independently following two stages: (1) selection by reading titles and abstracts; (2) selection by reading full texts. In each stage, the selection lists were cross checked, and in cases of discrepancies, a third reviewer participated in the process. In the second stage, reasons for exclusion were noted.

### 2.6. Data Extraction Process and Data Items

Data extraction of included studies was conducted independently for two researchers using an Excel^®^ spreadsheet (Microsoft Excel, version 16.0, Redmond, Washington, DC, USA). The data extraction form was adapted from the template provided on the Joanna Briggs Institute website. Prior to its use, the form underwent a pilot phase to assess the need for any refinements. In cases where studies authored by the researchers were evaluated, data extraction was conducted by independent external reviewers (M.M. and A.E.-H.) with no involvement in the primary studies.

Data were sought for the following variables: (1) article identification (authors, year of publication, country of origin of the first author, title, journal, and language), (2) study design, (3) study objective, (4) type of presentation of CBD, (5) evaluation test (e.g., minimum inhibitory concentration (MIC) tests), (6) contact method (e.g., eluate, dilutions, etc.), (7) controls, (8) evaluation time of the test, (9) primary outcome, and (10) sponsorship. The primary outcome was defined based on the main objective of the study in cases where it was not directly reported by the authors. Any data not found or reported were categorized as not reported (N/R).

In cases where clarification was needed, especially for studies that did not clearly report results or were not freely accessible, the authors of the articles were contacted via email or social media, with at least one attempt made to obtain further information.

### 2.7. Critical Appraisal of Individual Sources of Evidence

Although one study involved the collection of dental plaque samples from humans, all antimicrobial measurements in the included studies were conducted in vitro. In this regard, the methodological quality and risk of bias in the included studies were assessed using the RoBDEMAT tool, adapted for laboratory-based studies [29]. This tool evaluated nine items related to various sources of bias across four domains: bias related to planning and allocation (D1), sample preparation (D2), outcome assessment (D3), and data processing and reporting of results (D4). Each item was classified as sufficiently reported, insufficiently reported, or not reported for all studies included in the systematic review. Two independent reviewers assigned ratings to the items, and any discrepancies were resolved through consultation with a third reviewer. This critical appraisal of individual sources of evidence was conducted to ensure that the studies’ methodological quality was properly assessed, with the findings used to inform the data synthesis process. Studies authored by any of the researchers were assessed by independent researchers with no involvement in the primary study to minimize the risk of bias.

### 2.8. Strategy for Data Synthesis

The synthesis of data was conducted descriptively, taking into account the significant heterogeneity in study designs, methodologies, and reported outcomes. For example, more than 10 microbial species have been analyzed in the literature using protocols that assess antibacterial activity qualitatively but with differing methodologies. Given this variability, a qualitative approach was adopted to organize the studies into thematic and methodological categories, rather than attempting a quantitative synthesis. Studies were grouped based on study type (in vitro or in vivo), microbiological focus (antibacterial properties, antifungal properties, or effects on multispecies biofilms), and methods used (minimum inhibitory concentration (MIC) tests, counting colony-forming units, direct contact tests, metabolic activity and biofilm formation assays, and transcriptomic analyses such as RNA-seq).

The results were synthesized to highlight key findings regarding the efficacy of CBD against specific pathogens (e.g., *S. mutans*, *P. gingivalis*, and *C. albicans*), comparisons with other antimicrobial agents (e.g., chlorhexidine), and proposed mechanisms of action (e.g., changes in gene expression and biofilm inhibition).

## 3. Results

### 3.1. Results-Selection of Sources of Evidence

**Figure 1:**
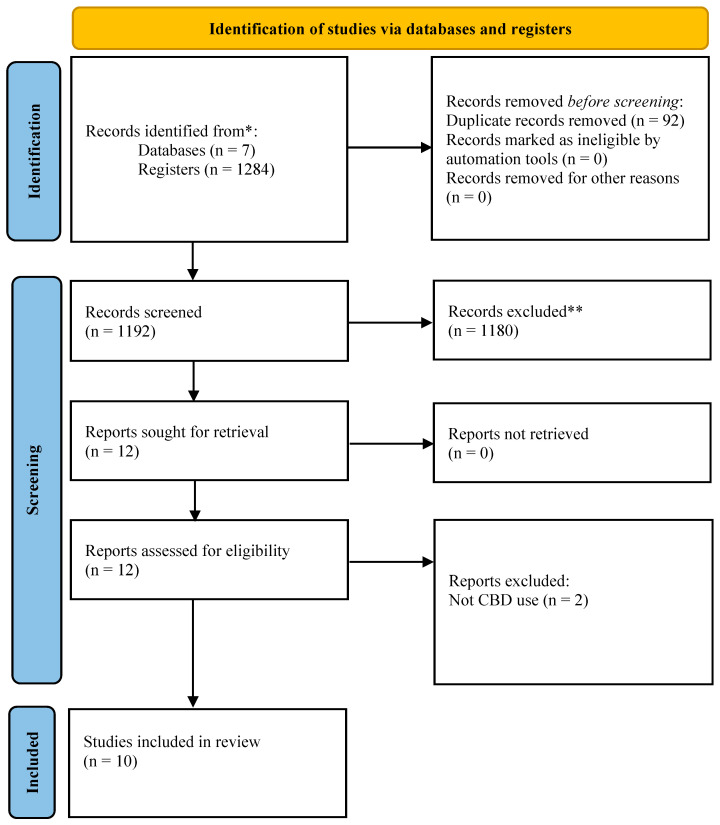
The PRISMA flow chart for reporting systematic reviews. * Consider, if feasible to do so, reporting the number of records identified from each database or register searched (rather than the total number across all databases/registers). ** If automation tools were used, indicate how many records were excluded by a human and how many were excluded by automation tools.

A total of 1284 articles were identified (Figure 1), with 1272 articles excluded based on their title and/or abstract. Among the 12 full-text articles assessed for eligibility, 10 studies were included in the qualitative synthesis in this review.

### 3.2. Characteristics of Sources of Evidence

Characteristics of the ten (100%) original studies included are presented in Table 3. In the percentage breakdown of the results, overlaps between categories were allowed, as a single study could be included in more than one of the analyzed categories. These studies were published between 2019 and 2025 in various journals, most of which were not exclusively dedicated to dental science. Based on the nationality of the first author, the countries that were published were Argentina, Venezuela, Israel, Colombia, the United States, the Czech Republic, Denmark, Belgium, and Brazil. The studies involved experiments in bacterial (90%) [30,31,32,33,34,35,36,37,38] or fungal cultures (20%) [38,39].

Seven studies (70%) used CBD of commercial origin as the active ingredient, five of them in the form of granules, one in the form of an extract, and one of them did not mention its form of presentation [31,32,33,35,36,38,39]. Three studies used CBD as the active ingredient, experimentally produced in the form of an extract [30,34], and one of them did not mention its form of presentation [37]. The most commonly used methods were the minimum inhibitory concentration test (50%) [33,34,36,37,38] and counting colony-forming units (50%) [32,33,35,36,37]. The methods for antimicrobial analysis were the direct contact test, the dilution test, the MIC test, counting colony-forming units, and RNAseq analysis. The methods for antifungal analysis were the minimum inhibitory concentration test, the metabolic activity test, the total biomass, and the biofilm/hyphae formation test. The method of contact between CBD and bacterial/fungal cell cultures was in dilution (90%) [30,31,32,33,34,35,36,37,38] or directly (30%) [30,36,39]. CBD activity was tested against *S. aureus* (30%) [30,31,34], *S. mutans* (20%) [33,38], *S. epidermidis* (10%) [34], *F. alocis* (10%) [32], *P. gingivalis* (20%) [32,33], *A. naeslundii* (10%) [37], P. anaerobius (10%) [37], *V. parvula* (10%) [37], *F. nucleatum* (10%) [37], *A. actinomycetemcomitans* (10%) [37], *P. aeruginosa* (10%) [34], *T. denticola* (20%) [32,35], *E. coli* (20%) [30,34], *C. albicans* (20%) [38,39], and in a multispecies biofilm (10%) [38]. Sponsors of the studies were either federal funding agencies, governmental authorities, universities, or private companies.

### 3.3. Critical Appraisal Within Sources of Evidence

The risk of bias analysis, conducted using the RoBDEMAT tool, revealed that among the ten included studies, one study met 100% of the proposed criteria [33]. Five studies met 70% of the criteria [30,31,36,38,39], with insufficient reporting of randomization and sample size selection, and no mention of blinding of the operators in the tests conducted. Four studies met 60% of the criteria [32,34,35,37], lacking sufficient reporting on group creation criteria, randomization, sample size selection, and statistical data analysis, as well as no mention of blinding of the operators in the tests (Table 4).

### 3.4. Synthesis of Results and Summary of Evidence

The studies included in this review suggest that CBD may have potential antimicrobial and antifungal properties. One study reported that MIC of CBD for *S. mutans* was 20 μg/mL [38]. Another study [33] found the MIC for *P. gingivalis* and *S. mutans* to be 1.5 μg/mL and 16 μg/mL, respectively, compared to 1 μg/mL for the control group. CBD demonstrated antimicrobial activity against Gram-positive bacteria, with MIC values ranging from 1 to 2 μg/mL, while cannabidiolic acid (CBDA) showed a two-fold lower activity. However, neither CBDA nor CBD showed antibacterial activity against any Gram-negative strains at concentrations of 64 μg/mL [34]. The MIC for different strains of periodontal bacteria when CBD was evaluated ranged from 0.39 to 3.12 μg/mL, compared to 0.23 to 1.84 μg/mL in the control group using chlorhexidine [37]. Furthermore, no MIC for *Candida albicans* was observed with CBD [38].

In the colony-forming unit (CFU) counting, one study reported that high doses of CBD effectively suppressed the growth of *P. gingivalis* and *F. alocis*, with no significant effect on the growth of *T. denticola* [32]. In the CBD group, a decreasing trend in bacterial count was observed for P. gingivalis when compared to the placebo and chlorhexidine groups, where the bacterial count remained similar over time [33]. Exposure to various concentrations of CBD (0–10 μg/mL) did not influence the growth of *T. denticola* ATCC 35405 [35]. When evaluating chlorhexidine (control group) and CBD (experimental group) on *A. naeslundii*, *P. anaerobius*, *V. parvula*, *F. nucleatum*, and *A. actinomycetemcomitans*, a decrease in bacterial growth was observed, especially as the concentrations of both agents increased [37]. Furthermore, CBD-infused mouthwash products showed comparable efficacy to chlorhexidine 0.2% in inhibiting bacterial growth from dental plaque samples obtained from humans [36].

In the direct contact test and dilution method, alginate scaffolds containing CBD extracts, as well as pure CBD extract, were more effective in inhibiting the growth of *Staphylococcus aureus* and *Escherichia coli*, compared to the control group and alginate scaffolds without CBD [30]. Another study using the direct contact test demonstrated that chlorhexidine exhibited higher antimicrobial activity against *S. aureus* at all times evaluated. Furthermore, hydrogel formulations containing PLGA@CBD particles, as well as PLGA@CBD particles alone, were more effective than the hydrogel group without PLGA@CBD particles, showing enhanced efficacy up to 12 and 48 h of evaluation [31].

For the metabolic activity, total biomass, and biofilm and hyphae formation test, the sustained-release varnish group with the addition of CBD and triclosan demonstrated greater activity against the growth of *C. albicans* compared to the varnish group with the addition of CBD or triclosan alone. The latter proved to be more effective compared to the control group [39]. For the antibiofilm activity test, multispecies biofilm metabolic activity was reduced by 50.38% with CBD at 125 μg/mL compared with a 90% reduction in the chlorhexidine treated group [38]. Finally, a transcriptomic study using RNA-seq identified a total of 392 *T. denticola* genes as CBD-responsive, consistent with findings from another study [35].

## 4. Discussion

This study presents new insights into the antimicrobial and antifungal properties of CBD, highlighting its potential as an alternative to traditional antimicrobial agents in oral health care. By synthesizing data from multiple studies, this review presents an evidence base for CBD therapeutic potential, paving the way for further research and clinical trials. Research to date has primarily investigated the antimicrobial activity of CBD against bacterial and fungal cultures using various methodologies, with the minimum inhibitory concentration test and counting colony-forming units being the most common. Thus, CBD can exhibit antimicrobial effects through mechanisms involving either dilution or direct interaction with microorganisms. The sources and concentrations of CBD used varied widely across the studies. This review provides an overview of CBD formulations, the microbial strains analyzed, and the broader challenges for advancing research in this field.

The studies evaluated the effectiveness of CBD as an antimicrobial agent using different assays. The diversity in methods and bacterial strains may influence the direct comparison of results. Regarding the concentrations and dosages in the CBD preparations used, the results show heterogeneity in both the methodologies employed and the mechanisms of active ingredient release. According to the results, the MIC test and the CFU counting assay were the most commonly used to evaluate the antimicrobial activity of CBD. This is consistent with studies examining the antimicrobial properties of other cannabinoids against microorganisms from the oral cavity [40] as well as other body regions [41,42]. The selection of this method allows for the analysis of the antibacterial capacity of active ingredients in solution, which is the most common form of CBD presentation in in vitro studies [40,41,42]. In this case, the broth dilution method is used, which, through serial dilutions of the active ingredient, enables the analysis of the effectiveness of different concentrations, specifically in the case of the MIC, to accurately assess its effectiveness [43]. These methods are those recommended by the literature and international organizations [43,44].

The doses tested varied across studies, depending on both the assay type and the CBD formulation used in the experiments. Furthermore, the results also differed according to the specific bacterial strains evaluated. The antimicrobial effectiveness of a commercial toothpaste containing CBD has been reported in the literature [45]. Similar outcomes were observed with toothpastes containing other cannabinoids, such as cannabinol (CBN), cannabigerol (CBG), and cannabichromene (CBC), which were evaluated against two commercial toothpastes [45,46]. In contrast, a mouthwash formulation containing 1% CBD was found to be effective against the growth of a dental plaque sample, producing a 1.5 cm inhibition halo, which was comparable to the positive control with chlorhexidine [36].

Other CBD delivery systems have also been evaluated. One study synthesized gelatin scaffolds with 0.066 μg/mL of CBD, suggesting inhibition of the growth of *S. aureus* and *E. coli* in the CBD groups compared to the groups without CBD [30]. In contrast, a hydrogel was synthesized with microparticles containing different concentrations of CBD (2.5%, 5%, and 10%), suggesting that against *S. aureus*, the CBD-containing groups were more effective than the groups without CBD, but not as effective as the positive control with chlorhexidine [31]. A sustained-release CBD varnish was synthesized, suggesting its effectiveness in reducing the biomass count of *C. albicans* compared to the group without CBD, with results similar to the control group with triclosan, where a synergistic effect between both was also observed [39].

Regarding the release mechanism of cannabinoids, the influence of recreational cannabis cigarette smoke on the oral microbiome has been analyzed, showing variations in the counts of the genera *Porphyromonas* and *Streptococcus* [47]. On the other hand, a study suggests that the consumption of medicinal cannabis cigarettes could lead to a decrease in *S. mutans* and an increase in the count of lactobacilli [48]. The differences in the results found could be due to the presentation, release mechanisms, and dosages analyzed [31,38,48]. Despite this, CBD has shown better results than other cannabinoids [33,45]. It seems that the antimicrobial efficacy of CBD also depends on the microorganism being analyzed [32,47].

Several strains of microorganisms present in the oral cavity have been analyzed, focusing on the microorganism involved in biofilms responsible for dental caries, such as *S. mutans* [22,23,33,38,40]. Other studies have also analyzed the effectiveness of cannabinoids on microorganisms associated with the onset of periodontal disease, such as *P. gingivalis*, *A. actinomycetemcomitans*, *F. alocis*, and *F. nucleatum*, suggesting a significant inhibitory effect on growth [32,33,42]. CBD has been shown to be an inhibitor of the growth of *C. albicans*, a type of commensal fungus in the oral cavity that causes candidiasis when various host protective factors are altered [35,49].

Limited literature attempts to explain the effect of CBD and other cannabinoids on oral pathogens suggest various mechanisms of action depending on the microorganism analyzed [30,31,32,33,34,35]. These mechanisms are crucial because they help clarify how CBD can be used as a targeted antimicrobial agent in oral healthcare, potentially improving the management of infections linked to dental diseases. Microscopy images of *S. mutans* indicate alterations in the cell wall structure, leading to a decrease in bacterial density. Biofilms of *S. mutans* treated with cannabinoids were more disorganized and exhibited greater internal porosity [23,40]. Such disorganization compromises the protective matrix of biofilms, making bacterial communities more susceptible to host defenses and other antimicrobial treatments [6,7]. For *S. aureus*, CBD may disrupt bacterial cytoplasmic membranes, inhibiting the synthesis of proteins, DNA, RNA, and peptidoglycans [30,31,50]. This multifaceted inhibition impairs essential bacterial functions like replication and metabolism, which could be valuable in preventing or treating oral infections caused by *S. aureus*. Cannabinoids also cause alterations in the cell membrane by inducing immediate hyperpolarization, increasing permeability, and promoting the accumulation of mesosome-like structures, leading to leakage of cellular contents and ultimately bacterial cell death [38,40].

Furthermore, CBD and other cannabinoids could inhibit the release of membrane vesicles and the quorum-sensing signal cascade involved in bacterial communication and interaction with the environment, regulating various functions, including biofilm formation, stress response, and virulence factor expression [33,39,51,52]. By interfering with quorum sensing, CBD can prevent bacteria from coordinating harmful activities such as biofilm maturation and expression of toxins, thus reducing pathogenicity and enhancing the effectiveness of conventional treatments [39,51,52]. In contrast, CBD suppresses the expression of *T. denticola* genes related to chemotaxis and proteolysis [35]. CBD and other cannabis components have been evaluated against *C. albicans*, suggesting they inhibit the adhesion of hyphae to epithelial cells, as well as yeast–hyphal transition and growth [38,49]. Since *C. albicans* is involved in oral candidiasis and biofilm-associated infections, these effects imply potential applications of CBD in antifungal therapies for oral mucosal infections [11,35,49]. In addition to its direct antimicrobial effects, CBD also exhibits notable anti-inflammatory activity and the ability to modulate the host immune response, which helps reduce local inflammation and promote the resolution of infectious processes in oral tissues, thereby enhancing its potential use in the treatment of microbially induced oral diseases [20,21].

In general, the presence and quantity of these microorganisms may increase in situations of microbiological imbalance or conditions that favor the proliferation of pathogens, such as periodontitis, diabetes, antibiotic use, or poor oral care. For this reason, it is important not to limit the study solely to the microorganisms responsible for the most prevalent oral diseases [30,31]. CBD and other cannabinoids have been shown to be effective as antimicrobials, in some cases with results similar to the controls (chlorhexidine, triclosan, and antibiotics) [23] and in other cases with results inferior to these [31]. Since natural products based on plant extracts may trigger fewer adverse effects compared to commercial products [53,54], they could be an alternative for use in dentistry.

Cannabinoids can be incorporated into biomaterials or controlled-release systems for various therapeutic purposes, such as optimizing their bioavailability, achieving sustained release, reducing systemic side effects, and thereby enhancing their efficacy [55,56]. These goals may vary depending on the area of application. For example, CBD and CBG have been integrated into 3D-printed sodium alginate films to increase contact time with tissues and promote the healing of soft-tissue lesions [57]. In another study, CBD-loaded liposomes were synthesized for the treatment of dentin hypersensitivity, demonstrating the ability to penetrate dentinal tubules, despite the hydrophilic nature of their interior and the lipophilicity of CBD [58]. Likewise, a porous hydrogel intended for intraosseous use and loaded with CBD was developed to promote bone regeneration, showing antimicrobial activity against *Staphylococcus aureus* [31]. Finally, CBD was incorporated into polymethyl methacrylate (PMMA), a material commonly used for complete dentures, demonstrating effectiveness against *C. albicans*, a pathogen frequently associated with denture-related stomatitis in prosthesis wearers [59].

In the current market, oral hygiene products based on plant extracts, which have been shown to be effective in controlling oral biofilms [60,61], are available. It is important to note that in recent decades, there has been an increase in the consumption of oral hygiene products [62] and that technological and industrial advancements have led to increased market competition. The addition of these agents to oral hygiene products, such as toothpastes and mouthwashes, could enhance their preventive effect against the most prevalent oral diseases [20,21]. The medicinal cannabis market has demonstrated sustained growth in terms of revenue [63,64]. This development has been driven by the expansion of legalization in various regions, as well as by the growing acceptance of cannabis as a therapeutic option [21,65,66].

However, these natural products can present challenges in meeting the quality standards required by published studies, as their preparation often results in variations in concentration and routes of administration [20,21]. One of the main challenges for the clinical integration of CBD is the significant variability in its availability, legal status, and quality control across different regions [65,66,67]. These disparities affect not only patients’ access to standardized and safe products but also the confidence of healthcare professionals in recommending their use. The lack of uniform regulations and the diversity of production standards can lead to inconsistencies in the composition and efficacy of commercially available CBD products [68]. Nevertheless, this scenario also presents an opportunity to develop clear regulatory frameworks and evidence-based clinical protocols that support the safe and effective use of CBD in dental and broader medical practice.

This study presents some limitations. Firstly, a significant number of the included studies used in vitro models, which may not accurately replicate the dynamic conditions of the oral environment. The interactions between cannabinoids, including CBD, and the oral microbiota have the potential to substantially influence its effects; however, these factors are not adequately represented in the in vitro settings used in the studies. However, we observed that CBD was effective against some oral pathogens. This lack of longitudinal studies prevents a comprehensive evaluation of CBD safety and efficacy in human populations under continuous use. Furthermore, there is variability in the composition of CBD-containing products with understanding long-term adverse effects or interactions with other treatments. Finally, the influence of external factors, such as diet, oral hygiene practices, and the concurrent use of other oral hygiene products, could affect the results. These variables are often not rigorously controlled in the studies, adding another layer of complexity to the interpretation of the findings.

However, this review has several positive and important points. Firstly, it used a comprehensive and rigorous literature search, incorporating both published and gray literature, thereby ensuring the inclusion of a broad and relevant range of articles in the qualitative synthesis. The data search and data extraction were performed by individuals who had undergone calibration tests, increasing the reliability of the study. In some studies, dental plaque samples were obtained directly from individuals. For this, it was possible to include different methodological approaches and evaluate different pathogens, including both Gram-positive and Gram-negative bacteria as well as fungi. This increases the generalizability of the findings. The comparison with traditional antimicrobial agents, like chlorhexidine, adds practical relevance, suggesting CBD could serve as an effective alternative or complement in dental treatments. Furthermore, the use of the RoBDEMAT tool to assess the quality of the included studies demonstrated the minimum expected standards for studies focused on dental materials. For this reason, future in vitro studies analyzing other experimental and commercial CBD products should consider standardizing the methodologies applied, clearly reporting aspects such as criteria for group creation, randomization, sample size selection, and statistical data analysis, among others. More studies evaluating clinical aspects such as inflammation and bleeding of the gums, presence of bacterial plaque, and state of carious lesions are needed, with long-term follow-up of individuals, analyzing different concentrations, administration routes, and their effects on the oral microbiota profile. The analysis of cannabinoid availability in saliva and blood could help determine the minimum effective concentrations.

## 5. Conclusions

The current knowledge regarding the microbiological properties of CBD indicates its antimicrobial potential against oral microorganisms such as *P. gingivalis*, *S. mutans*, and *C. albicans*. Several studies have evaluated CBD antimicrobial effects through assays such as the MIC test and bacterial growth assays, with varying concentrations and formulations. These studies suggest that CBD can inhibit microbial growth, though its effectiveness varies according to CBD concentration, microbial strain, and the delivery system. However, due to the diversity of methodologies, CBD formulations, and bacterial strains evaluated, further clinical studies are needed to establish standardized protocols and better understand the full range of CBD antimicrobial capabilities in oral health.

## Figures and Tables

**Table 1:** Eligibility criteria for included studies.

Inclusion Criteria	Exclusion Criteria
- Studies exploring the effects of CBD on oral microbiology (in vitro and in vivo in animal models).	- Studies not addressing the microbiological properties of CBD.
- Studies examining CBD properties when isolated or combined with other substances.	- Case reports, case series, and pilot studies.
- Studies published in peer-reviewed scientific journals.	- Opinion articles, letters, conference abstracts, and review articles.
- No restrictions on language or year of publication.	- Studies related to the use or smoking of cannabis plants and other cannabinoids.
	- Studies focusing on other antibacterial agents (e.g., triclosan, chlorhexidine, and pyridinium acetate).
	- Studies involving pathogens not belonging to the oral cavity.
	- Studies focusing exclusively on salivary characteristics (such as quantity or pH).

**Table 2:** Combination of medical subject headings (MeSHs) along with free-text words used and adapted in each of the selected databases.

Section	Terms
#1: Terms related to cannabis	(Cannabis) OR (Cannabidiol) OR (Medical Marijuana) OR (Marijuana, Medical) OR (Medicinal Cannabis) OR (Cannabis, Medicinal) OR (Marijuana Treatment) OR (Treatment, Marijuana) OR (Medicinal Marijuana) OR (Marijuana, Medicinal) OR (Medical Cannabis) OR (Cannabis, Medical) OR (Marijuana Dispensaries) OR (Dispensaries, Marijuana)
#2: Terms related to dentistry	(Dentistry) OR (Mouth) OR (Oral Health) OR (Dental)
#3: Terms related to antimicrobials and biofilms	(Antimicrobial) OR (Biofilms) OR (Fungi) OR (Mycoses) OR (Anti-Infective Agents) OR (Anti-Bacterial Agents) OR (Toothpastes) OR (Mouthwashes)
#1 AND #2 AND #3: Combined terms	All

**Table 3:** Characteristics of the original articles included (n = 10).

Article Identification				Study Design	Type of Presentation of CBD	Evaluation Method	Contact Method	Control	MO	Time	Primary Outcome	Funding
Author and Year	Country	Title	Journal and Language									
Antezana 2022 [30]	Argentina	Design of a New 3D Gelatin—Alginate Scaffold Loaded with Cannabis sativa Oil	*Polymers* English	In vitro	Experimental—oil extract	DCT (the solid and the dilution method)	Direct (IH DLBM)	Group without CBD	*S. aureus*; *E. coli*	12–24 h	Alginate scaffolds with CBD extracts were effective against the growth of *S. aureus* and *E. coli*.	CONICET, UBACYT, and PIDAE, Argentina
David 2024 [31]	Venezuela	Cannabidiol-loaded microparticles embedded in a porous hydrogel matrix for biomedical applications	*J. Mater. Sci. Mater. Med.* English	In vitro	Commercial—oil extract	DCT (dilution method)	Indirect (dilution in PBS)	Positive control: CHX Negative control: Distilled water	*S. aureus*	504 h	Hydrogel groups with PLGA@CBD particles and PLGA@CBD particles alone were shown to be effective against *S. aureus*.	CAPES and FAPERGS, Brazil
Feldman 2022 [39]	Israel	Potential Combinatory Effect of Cannabidiol and Triclosan Incorporated into Sustained Release Delivery System against Oral Candidiasis	*Pharmaceutics* English	In vitro/ex vivo *model*	Commercial—N/S	Metabolic activity, total biomass, biofilm hyphae formation	Direct	Group without CBD and triclosan	*C. albicans*	336 h	The sustained-release varnish group with the addition of CBD and triclosan demonstrated greater activity against the growth of *C. albicans*.	N/S
Garzón 2024 [38]	Colombia	Antibiofilm and Immune-Modulatory Activity of Cannabidiol and Cannabigerol in Oral Environments—In Vitro Study	*Antibiotics* English	In vitro	Commercial—powder	MIC Antibiofilm activity (2,3,5-triphenyltetrazolium chloride- TTC)	Indirect (Dilution in PBS)	Positive control: CHX amphotericin B and fluconazole	*S. mutans*; *C. albicans*; multispecies biofilm	MIC: 27 antibiofilm activity: 168 h	MIC for *S. mutans* with CBD was 20 μM. Multispecies biofilm metabolic activity was reduced by 50.38% with CBD at 125 μg/mL.	The Pontificia Universidad Javeriana, Colombia
Gu 2019 [32]	United States	Marijuana-Derived Cannabinoids Trigger a CB2/PI3K Axis of Suppression of the Innate Response to Oral Pathogens	*Front Immunol.* English	In vitro	Commercial—powder	CFU counting	Indirect (dilution in solvents)	Group without CBD	*F. alocis*; *P. gingivalis*; *T. denticola*	40, 80, and 250 h	CBD suppressed the growth of *P. gingivalis* and *F. alocis*.	NIDCR, USA
Jirasek 2024 [33]	Czech Republic	Phytocannabinoids and gingival inflammation: Preclinical findings and a placebo-controlled double-blind randomized clinical trial with cannabidiol	*J. Periodontal Res.* English	*Clinical*/In vitro	Commercial—powder	MIC; CFU counting	Indirect (dilution in gel and toothpaste)	Positive control: CHX Group without CBD	*P. gingivalis*, *S. mutans*	168, 672, and 1344 h	MIC for *P. gingivalis* and *S. mutans* with CBD was 1.5 and 16 μg/mL, respectively. CBD decreasing trends in bacterial count.	CB21 Pharma Ltd., CBDepot Ltd., and PharmaCan Ltd., Czech Republic
Martinenghi 2020 [34]	Denmark	Isolation, Purification, and Antimicrobial Characterization of Cannabidiolic Acid and Cannabidiol from Cannabis sativa L.	*Biomolecules* English	In vitro	Experimental—oil extract	MIC	Dilution in methanol	Positive control: CLI, OFX, MEM, and TOB	*S. aureus*; *S. epidermidis*, *E. coli*, and *P. aeruginosa*	24 h	CBD demonstrated a potent activity against Gram-positive bacteria with a minimal inhibitory concentration between 1 and 2 μg/mL.	The Danish Council for Independent Research, Denmark
Tan 2024 [35]	United States	The transcriptomic response to cannabidiol of Treponema denticola, a phytocannabinoid-resistant periodontal pathogen	*J. Clin. Periodontol.* English	In vitro	Commercial—powder	CFU counting; RNAseq analysis	Dilution in methanol	Group without CBD	*T. denticola*	240 h	Growth of *T. denticola* was not influenced by exposure to various concentrations of CBD (0–10 μg/mL).	University of Louisville, USA
Vasudevan 2020 [36]	Belgium	Cannabinoids infused mouthwash products are as effective as chlorhexidine on inhibition of total-culturable bacterial content in dental plaque samples	*J. Cannabis Res.* English	*Clinical*/In vitro	Commercial—powder.	MIC; CFU counting	IH DLBM	Positive group: CHX	NS	36 h	CBD-infused mouthwash products inhibit bacterial growth.	VLAIO Belgium/CannIBite bvba
Santos 2024 [37]	Brazil	The antibacterial and antibiofilm role of cannabidiol against periodontopathogenic bacteria	*J Appl Microbiol.* English.	In vitro	Experimental—N/S	MIC; CFU counting	Dilution in DMSO	Positive group: CHX Group without CBD	*A. naeslundii*; *P. anaerobius*; *V. parvula*; *F. nucleatum*; *A. actinomycetemcomitans*	24, 36, 48, 72, 96, and 168 h	MIC for CBD in the bacteria tested ranged from 0.39 to 3.12 μg/mL. CBD decreasing trends in bacterial count.	Ministry of Education of the Brazil (MEC) and EMBRAPII

CBD: cannabidiol; CBDA: cannabidiolic acid; CFUs: colony-forming units; CHX: chlorhexidine; CLI: clindamycin; OFX: ofloxacin; MEM: meropenem; TOB: tobramycin; DCT: direct contact test; PBS: phosphate-buffered saline; IH: inhibition halo; DLBM: dilution in Luria-Bertani medium; DMSO: dimethyl sulfoxide; MIC: minimum inhibitory concentration; MO: microorganism; CONICET: National Scientific and Technical Research Council; UBACYT: University of Buenos Aires Scholarship; PIDAE: Research and Development Projects in Strategic Areas with Social Impact; CAPES: Coordination for the Improvement of Higher Education Personnel; FAPERGS: Rio Grande do Sul Research Foundation; NIDCR: National Institute of Dental and Craniofacial Research; EMBRAPII: Brazilian Company of Research and Industrial Innovation; NS: not specified.

**Table 4:** RobDEMAT criteria of included articles.

	Bias in Planning and Allocation			Bias in Sample/Specimen Preparation			Bias in Outcome Assessment		Bias in Data Treatment and Outcome Reporting		
	Control Group	Randomization of Samples	Sample Size Rationale and Reporting	Standardization of Samples and Materials	Identical Experimental Conditions Across Groups	Identical Experimental Conditions Across groups	Adequate and Standardized Testing Procedures and Outcome	Blinding of the Test Operator	Statistical Analysis	Reporting Study Outcomes	Total Criteria Met (%)
Antezana, 2022 [30]	YES	NO	NO	YES	YES	YES	YES	NR	YES	YES	70%
David, 2024 [31]	YES	NO	NO	YES	YES	YES	YES	NR	YES	YES	70%
Feldman, 2022 [39]	YES	NO	NO	YES	YES	YES	YES	NR	YES	YES	70%
Garzón, 2024 [38]	YES	NO	NO	YES	YES	YES	YES	NR	YES	YES	70%
Gu, 2019 [32]	NO	NO	NO	YES	YES	YES	YES	NR	YES	YES	60%
Jirasek, 2024 [33]	YES	YES	YES	YES	YES	YES	YES	YES	YES	YES	100%
Martinenghi, 2020 [34]	YES	NO	NO	YES	YES	YES	YES	NR	NO	YES	60%
Tan, 2024 [35]	YES	NO	NO	YES	YES	YES	YES	NR	NO	YES	60%
Vasudevan, 2020 [36]	YES	NO	NO	YES	YES	YES	YES	NR	YES	YES	70%
Santos, 2024 [37]	YES	NO	NO	YES	YES	YES	YES	NR	NR	YES	60%

Code. YES: sufficiently reported; NO: insufficiently reported; NR: no report.

## Data Availability

No new data were created or analyzed in this study. Data sharing is not applicable to this article.

## Supplementary Materials

The following supporting information can be downloaded at: https://www.mdpi.com/article/10.3390/dj13110519/s1. Table S1: Search strategy used in the different databases.

## Abbreviations

The following abbreviations are used in this manuscript:

CBD	Cannabidiol
CBDA	Cannabidiolic acid
CFUs	Colony-forming units
CHX	Chlorhexidine
CLI	Clindamycin
OFX	Ofloxacin
MEM	Meropenem
TOB	Tobramycin
DCT	Direct contact test
PBS	Phosphate-buffered saline
IH	Inhibition halo
DLBM	Dilution in Luria–Bertani medium
DMSO	Dimethyl sulfoxide
MIC	Minimum inhibitory concentration
MO	Microorganism
PMMA	Polymethylmethacrylate
