# Supplementary material for: Potential Antimicrobial Use of Cannabidiol in Dentistry: A Scoping Review
Source: Dent J (Basel). 2025 Nov 6;13(11):519. doi: 10.3390/dj13110519 (PMC12651879; doi:10.3390/dj13110519)
Supplement: Supplementary file 1 [file dentistry-13-00519-s001.zip › dentistry-3654834-supplementary.pdf]

Table Supplementary 1. Search strategy used in the different databases.

PubMed/MEDLINE	(Cannabis) OR (Cannabidiol) OR (Medical Marijuana) OR (Marijuana, Medical) OR (Medicinal Cannabis) OR (Cannabis, Medicinal) OR (Marijuana Treatment) OR (Treatment, Marijuana) OR (Medicinal Marijuana) OR (Marijuana, Medicinal) OR (Medical Cannabis) OR (Cannabis, Medical) OR (Marijuana Dispensaries) OR (Dispensaries, Marijuana) AND (Dentistry) OR (Mouth) OR (Oral Health) OR (Dental) AND (Antimicrobial) OR (Biofilms) OR (Fungi) OR (Mycoses) OR (Anti-Infective Agents) OR (Anti-Bacterial Agents) OR (Toothpastes) OR (Mouthwashes)
Cochrane Library	(Cannabis) OR (Cannabidiol) OR (Medical Marijuana) OR (Marijuana, Medical) OR (Medicinal Cannabis) OR (Cannabis, Medicinal) OR (Marijuana Treatment) OR (Treatment, Marijuana) OR (Medicinal Marijuana) OR (Marijuana, Medicinal) OR (Medical Cannabis) OR (Cannabis, Medical) OR (Marijuana Dispensaries) OR (Dispensaries, Marijuana) AND (Dentistry) OR (Mouth) OR (Oral Health) OR (Dental) AND (Antimicrobial) OR (Biofilms) OR (Fungi) OR (Mycoses) OR (Anti-Infective Agents) OR (Anti-Bacterial Agents) OR (Toothpastes) OR (Mouthwashes)
Scopus	ALL (“Cannabis“ OR “Cannabidiol“ OR “Medical Marijuana“ OR “Marijuana, Medical“ OR “Medicinal Cannabis“ OR “Cannabis, Medicinal“ OR “Marijuana Treatment“ OR “Treatment, Marijuana“ OR “Medicinal Marijuana“ OR “Marijuana, Medicinal“ OR “Medical Cannabis“ OR (Cannabis, Medical“ OR “Marijuana Dispensaries“ OR “Dispensaries, Marijuana““ AND (“Dentistry“ OR “Mouth“ OR “Oral Health“ OR “Dental“) AND (“Antimicrobial“ OR “Biofilms“ OR “Fungi“ OR “Mycoses“ OR “Anti-Infective Agents“ OR “Anti-Bacterial Agents“ OR “Toothpastes“ OR “Mouthwashes“)
Embase	(Cannabis) OR (Cannabidiol) OR (Medical Marijuana) OR (Marijuana, Medical) OR (Medicinal Cannabis) OR (Cannabis, Medicinal) OR (Marijuana Treatment) OR (Treatment, Marijuana) OR (Medicinal Marijuana) OR (Marijuana, Medicinal) OR (Medical Cannabis) OR (Cannabis, Medical) OR (Marijuana Dispensaries) OR (Dispensaries, Marijuana) AND (Dentistry) OR (Mouth) OR (Oral Health) OR (Dental) AND (Antimicrobial) OR (Biofilms) OR (Fungi) OR (Mycoses) OR (Anti-Infective Agents) OR (Anti-Bacterial Agents) OR (Toothpastes) OR (Mouthwashes)
Web Of Science	TS = (Cannabis) OR (Cannabidiol) OR (Medical Marijuana) OR (Marijuana, Medical) OR (Medicinal Cannabis) OR (Cannabis, Medicinal) OR (Marijuana Treatment) OR (Treatment, Marijuana) OR (Medicinal Marijuana) OR (Marijuana, Medicinal) OR (Medical Cannabis) OR (Cannabis, Medical) OR (Marijuana Dispensaries) OR (Dispensaries, Marijuana) AND (Dentistry) OR (Mouth) OR (Oral Health) OR (Dental) AND (Antimicrobial) OR (Biofilms) OR (Fungi) OR (Mycoses) OR (Anti-Infective Agents) OR (Anti-Bacterial Agents) OR (Toothpastes) OR (Mouthwashes)
SciELO	((Cannabis) OR (Cannabidiol) OR (Medical Marijuana) OR (Marijuana, Medical) OR (Medicinal Cannabis) OR (Cannabis, Medicinal) OR (Marijuana Treatment) OR (Treatment, Marijuana)) AND ((Dentistry) OR (Mouth) OR (Oral Health) OR (Dental)) AND (Antimicrobial) OR (Biofilms) OR (Fungi) OR (Mycoses) OR (Anti-Infective Agents) OR (Anti-Bacterial Agents) OR (Toothpastes) OR (Mouthwashes))
Lilacs	((Cannabis) OR (Cannabidiol) OR (Medical Marijuana) OR (Marijuana, Medical) OR (Medicinal Cannabis) OR (Cannabis, Medicinal) OR (Marijuana Treatment) OR (Treatment, Marijuana)) AND ((Dentistry) OR (Mouth) OR (Oral Health) OR (Dental)) AND (Antimicrobial) OR (Biofilms) OR (Fungi) OR (Mycoses) OR (Anti-Infective Agents) OR (Anti-Bacterial Agents) OR (Toothpastes) OR (Mouthwashes)
